# Identification of signature genes associated with therapeutic resistance to anti-VEGF therapy

**DOI:** 10.18632/oncotarget.27307

**Published:** 2020-01-07

**Authors:** Pharavee Jaiprasart, Samrita Dogra, Deepika Neelakantan, Bharat Devapatla, Sukyung Woo

**Affiliations:** ^1^ Department of Pharmaceutical Sciences, College of Pharmacy, the University of Oklahoma Health Sciences Center, Oklahoma City, Oklahoma, USA; ^2^ Gynecologic Cancers Research Program, Peggy and Charles Stephenson Cancer Center, the University of Oklahoma Health Sciences Center, Oklahoma City, Oklahoma, USA

**Keywords:** anti-VEGF resistance, angiogenesis, bevacizumab, next-generation sequencing (NGS), apelin

## Abstract

VEGF-mediated tumor angiogenesis is a validated clinical target in many cancers, but modest efficacy and rapid development of resistance are major challenges of VEGF-targeted therapies. To establish a molecular signature of this resistance in ovarian cancer, we developed preclinical tumor models of adaptive resistance to chronic anti-VEGF treatment. We performed RNA-seq analysis and reverse-phase protein array to compare changes in gene and protein expressions in stroma and cancer cells from resistant and responsive tumors. We identified a unique set of stromal-specific genes that were strongly correlated with resistance phenotypes against two different anti-VEGF treatments, and selected the apelin/APJ signaling pathway for further *in vitro* validation. Using various functional assays, we showed that activation of apelin/APJ signaling reduces the efficacy of a VEGF inhibitor in endothelial cells. In patients with ovarian cancer treated with bevacizumab, increased expression of apelin was associated with significantly decreased disease-free survival. These findings link signature gene expressions with anti-VEGF response, and may thus provide novel targetable mechanisms of clinical resistance to anti-VEGF therapies.

## INTRODUCTION

Ovarian cancer is the principal cause of gynecological-cancer-related deaths in women in the United States [1]. Ovarian tumors are supported by a complex tumor microenvironment, are richly vascularized, and a significant correlation exists between microvascular count and biological aggressiveness [2, 3]. Increased tumor angiogenesis in ovarian cancer is also critical to ovarian cancer metastasis and ascites development [4]. Several studies have shown that a high level of vascular endothelial growth factor (VEGF), a key regulator of tumor angiogenesis, is associated with poor prognosis in patients with ovarian cancer [5, 6]. Accordingly, therapeutics targeting VEGF angiogenic pathways, such as the monoclonal antibody bevacizumab, have demonstrated clinical efficacy in combination with standard therapeutics; reducing the risk of disease worsening or death by 62%, compared with chemotherapy alone [7, 8]. Several phase III clinical studies have also shown that VEGF receptor (VEGFR) tyrosine kinase inhibitors (TKIs) significantly increase progression-free survival (PFS) when used as maintenance therapy [9]. Together, these data suggest that anti-angiogenic strategies are a valid and important treatment option for ovarian cancer. However, the clinical benefit from anti-VEGF therapy is transient, with rapidly emerging resistance as a major impediment [10]. Hence, there is an urgent need to identify biological markers implicated in the resistance to anti-VEGF drugs, which would aid not only in the early detection of resistance development, but also to monitor responders. The identified markers would also provide important alternative therapeutic strategies to improve the clinical benefit of anti-angiogenic drugs.

In the present study, we developed preclinical xenograft models of ovarian cancer that acquire adaptive resistance to anti-VEGF therapeutics. Using these tumors, we utilized unbiased approaches to identify not only a distinct gene signature associated with resistance development, but also the source of resistance (cancer cells versus tumor microenvironment). We further validated the role of some proteins as potential mechanisms of cancer resistance to anti-angiogenic therapy. This study provides avenues to identify novel underlying mechanisms of clinical resistance to anti-VEGF therapies, which when used in new combination strategies, may aid in counteracting these bypass mechanisms.

## RESULTS

### Development of tumors resistant to anti-VEGF therapy

**Figure 1:**
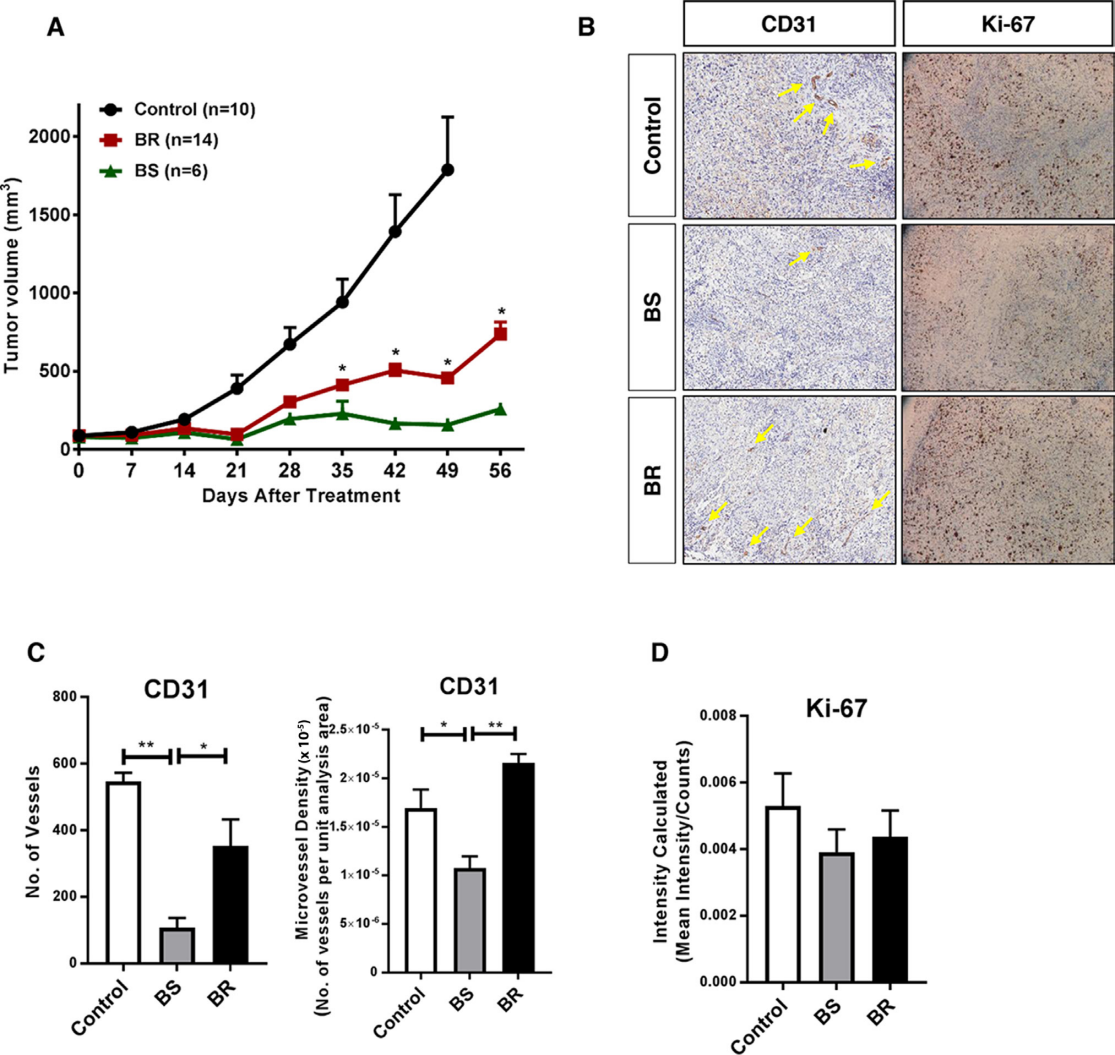
Adaptive resistance to anti-VEGF therapy in a xenograft mouse model of ovarian cancer **(A)** SKOV-3 xenograft-tumor bearing mice were chronically treated with bevacizumab (10 mg/kg, i.p., twice a week) or vehicle for two months. By the end of treatment, 14 of 20 (74%) mice showed tumor progression over bevacizumab treatment (BR - bevacizumab resistant) and the rest remained responsive (BS - bevacizumab sensitive). The control mice received vehicle treatment for the duration of the study. **(B)** Representative images of immunostaining for CD31 (micro vessel density; yellow arrows point to vessels) and Ki-67 (proliferation) in control, BS, and BR tumors. **(C, D)** Quantification of **(C)** CD31 expressed as number of vessels and microvessel density, and **(D)** Ki-67 in control, BS, and BR tumors. Statistical analysis was performed using two-way ANOVA for A, and one-way ANOVA for **C., D.** ^*^P<0.05, ^**^P<0.01.

To identify the molecular changes in ovarian tumors that progress despite anti-VEGF therapy, we developed a preclinical model for adaptive resistance, wherein the tumor-bearing mice received anti-VEGF treatment for two months. We used two different inhibitors against the VEGF pathway: bevacizumab, the monoclonal antibody that blocks the ligand VEGF secreted by the tumor cells, and sorafenib, a small-molecule TKI that targets VEGFR expressed by both endothelial cells (in the stroma) and cancer cells. The tumors that initially responded and then regrew while receiving continuous treatment of either drug (i.e., that developed adaptive resistance to the therapy) were designated as bevacizumab- or sorafenib-resistant (BR or SR). The tumors that remained stable in size with continued treatment were deemed bevacizumab- or sorafenib-sensitive (BS or SS). We also had a subgroup of control tumors from vehicle-treated mice at study endpoint. We observed an apparent trend of tumor regrowth in the resistant tumors starting at week 4-5 of treatment. By the end of the 8-week treatment period, we observed that around 70% of tumors had progressed despite bevacizumab treatment (Figure 1A), while 50% of tumors progressed despite sorafenib treatment (Supplementary Figure 1) [11]. Immunohistochemistry (IHC) analyses revealed an increase in CD31 staining in BR tumors compared with BS tumors (Figure 1B), depicted by a significant increase in both the number of vessels as well as microvessel density (MVD), and similar to that in control tumors (Figure 1C). BR tumors also had increased Ki-67 staining indicating increased proliferation, but the results were not statistically significant (Figure 1B, 1D). Together, these data show that the tumors that gained adaptive resistance with restored tumor progression, resumed angiogenesis and cell proliferation despite continued anti-VEGF treatment.

### Profiling treatment-specific changes in anti-VEGF-treated tumors

**Figure 2:**
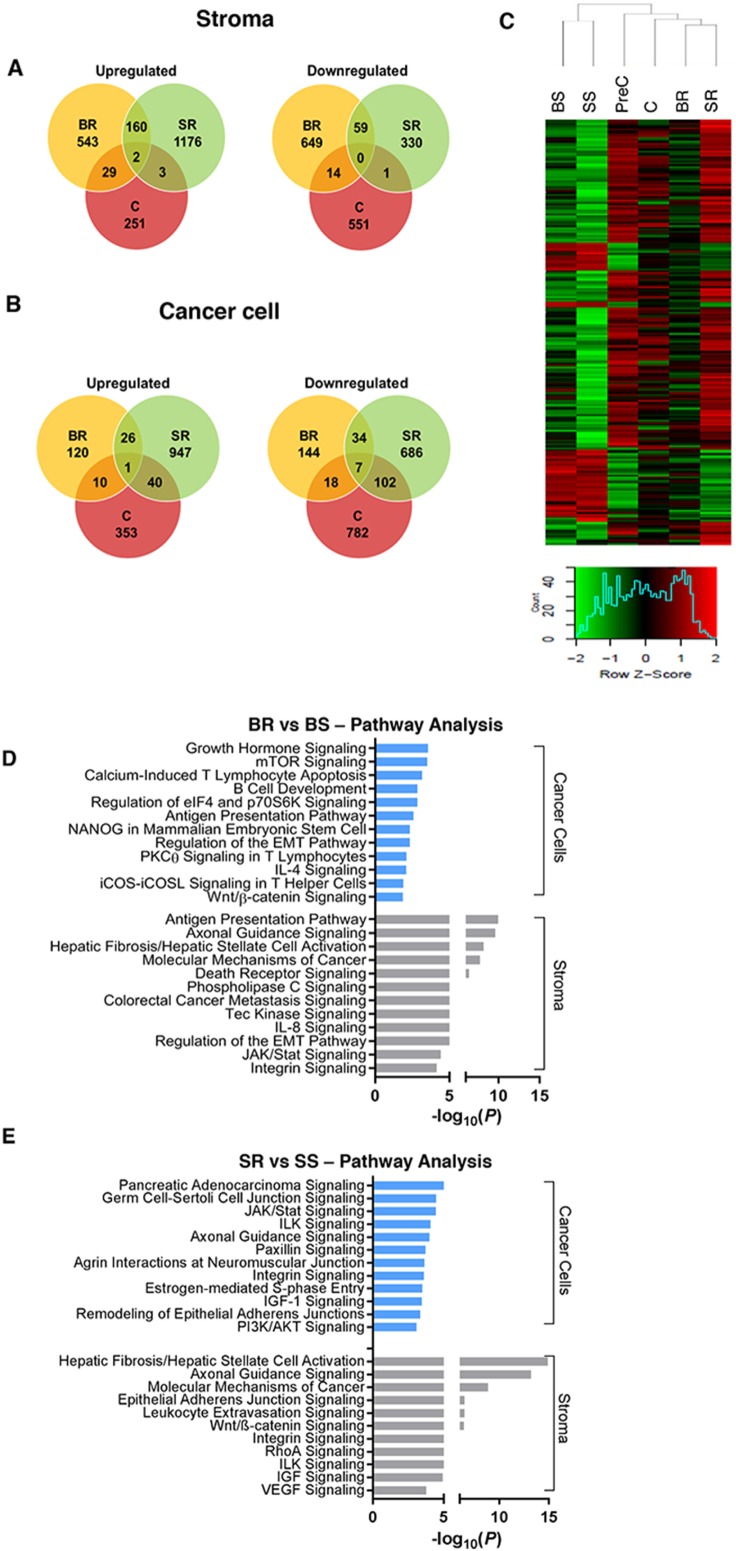
Analysis of gene expression changes in anti-VEGF resistant tumors **(A, B)** Venn diagrams showing unique and common genes differentially expressed in BR vs. BS, SR vs. SS, and C vs. PreC (p<0.05, FC>1.5) in **(A)** stroma or **(B)** cancer cells. **(C)** Heat map and hierarchical clustering of log2-transformed mRNA expression level of the top 200 differentially expressed stromal genes (ANOVA, p < 0.05) from RNA-seq analysis. **(D, E)** Most significantly pertinent canonical signal transduction pathways predicted by Ingenuity Pathway Analysis based on the differentially expressed genes between **(D)** BR vs. BS tumors and **(E)** SR vs. SS tumors; p<0.05. Statistical analysis was performed using ANOVA for **C.** and Fisher’s exact test for **D., E.** ^*^P<0.05.

To profile transcriptome changes due to anti-VEGF therapeutics, we collected tumors and performed RNA-seq analysis in the different treatment groups: pre-control (PreC, control tumors before treatment initiation), control (C, vehicle-treated control tumors at study endpoint), BR, BS, SR, and SS tumors. We identified genes that were at least 1.5-fold differentially regulated in resistant tumors compared with responsive tumors in each treatment group. The genes were further stratified based on whether they were expressed by cancer cells (human-originated) or by tumor stroma (mouse-originated). Supplementary Tables 1 and 2 list the top differentially expressed cancer-associated and stromal genes (BR/BS and SR/SS), respectively. More stromal genes (*n*=1456) than cancer cell-genes (*n*=360) were differentially regulated in BR tumors, whereas similar numbers of stromal (*n*=1731) and cancer cell-related genes (*n*=1753) were affected in SR tumors (Figure 2A-2B). This treatment-related difference is in line with their main sites of action, with bevacizumab mainly targeting the stroma, and sorafenib targeting both the stroma *and* cancer cells.

Among the cancer cell-originated genes, a small number (*n*=68) were common to BR and SR tumors. Numerous stroma-originating genes (*n*=221) were commonly upregulated (*n*=162) or downregulated (*n*=59) in the two treatment-resistant tumor groups. Of note, these common genes rarely overlapped with those in the vehicle-treated control tumors (large tumors denoted as C; Figure 2A-2B), despite similar Ki-67 and CD31 levels (Figure 1B), indicating that these transcriptional changes in the resistant tumors are treatment-related. Unsupervised hierarchical clustering also revealed distinct gene expression patterns, likely reflecting their resistance phenotypes (Figure 2C). For example, gene expression patterns were most similar among the sensitive tumors (BS and SS).

Next, we used Ingenuity Pathway Analysis (IPA) software to identify signaling pathways that were enriched in resistant tumors. In the BR and SR cancer cells, we observed several frequently activated pathways, such as the mTOR, Wnt, JAK/Stat, and PI3K/AKT signaling pathways (Figure 2D, 2E), that have been shown to increase cell survival and proliferation [12]. Using reverse-phase protein array (RPPA), we were able to validate our RNAseq data to confirm the upregulation of protein levels of some of the differentially regulated genes, including AKT and mTOR pathways in resistant tumors (Supplementary Figure 2A, 2B). Interestingly, VEGFR-2 was significantly upregulated in both BR and SR tumors. This finding is consistent with other studies glioblastoma, wherein tumors resistant to anti-angiogenic therapies targeting the VEGF pathway compensate by ramping up production of the receptor [13, 14]. In the stroma of BR tumors, we found upregulation of several pathways involved in immune response including the antigen presentation pathway, in addition to upregulation of EMT pathways, and those involved in cell-cell or cell-extracellular matrix interactions, such as the integrin signaling pathways (Figure 2D). Similar signaling cascades involved in cell-cell adhesion were highly activated in the stroma of SR tumors, in addition to axes such as the ILK and IGF signaling pathways (Figure 2E). Although not shown, frequently upregulated pathways such as the IL-8 and VEGF pathways were also activated in both the BR and SR tumor stroma, similar to what we previously reported [11]; together indicating that our IPA core analysis successfully identified several signal transduction pathways involved in cancer progression. The differential expression of the genes and proteins in the tumor groups suggest that the elevated expression of some of them may be involved in emergence of resistance phenotype to anti-VEGF therapy. As there were more common stromal gene signatures (*n*=221) between the BR and SR tumors (due to the agents being VEGF pathway-targeting drugs) we focused on identifying resistance-specific genes expressed by the tumor stroma.

### Identification of stroma-specific genes distinguishing resistant tumor phenotypes

**Figure 3:**
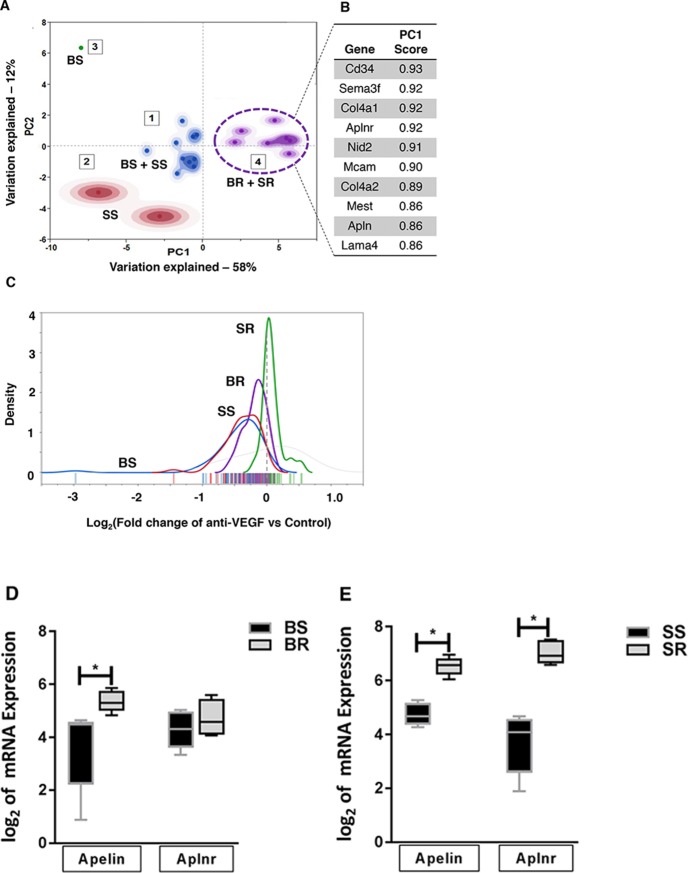
PCA analysis of stromal genes associated with resistant tumor phenotype **(A)** Principal component analysis-transformed data of stroma-originated genes differentiates resistance tumor phenotypes (BR+SR; cluster 4) from sensitive phenotypes (BS+SS; clusters 1-3). **(B)** Top ten genes with highest contribution to observed resistance phenotypes as identified by PCA in **A.** **(C)** Density plot of expression levels (log_2_fold change) of the top ten validated genes in all phenotypes. **(D, E)** Stromal mRNA expression of apelin (*Apln*) and APJ (*Aplnr*) in **(D)** BR and BS tumors and **(E)** SR and SS tumors. Statistical analysis was performed using Multiple t test ^*^P<0.05.

To ensure the clinical and biological significance of resistance-specific candidate genes, we focused on 30 genes that were also part of the VEGF-dependent vascular signaling signature (i.e., VDV genes) [15]. These VDV genes have been shown to be markedly downregulated in response to acute anti-VEGF treatment, and have been previously validated in human cancers [15]. Principal component analysis (PCA) of the candidate genes indicated that the gene expression profiles associated with resistant tumors (SR- and BR-tumors; Cluster 4) were distinct from the gene expression profiles associated with the sensitive tumors (SS- and BS-tumors; Clusters 1-3) (Figure 3A). We found that *Cd34, Sema3f, Col4a1*, *Col4a2, Aplnr, Nid2, Mcam, Mest, Apln*, and *Lama4* were among the top enriched genes in the mouse stroma that correlated most with the resistance phenotypes (Figure 3B). The transcriptional response of the top identified genes showed that post 2-month anti-VEGF treatment, their gene expressions were increased in the BR and SR tumors, compared with their respective control tumors (BS and SS; Figure 3C). Together, these data demonstrate that regulation of the top identified genes is closely related to resistance to anti-VEGF therapy.

Of the top enriched genes associated with the resistance phenotype, several such as *COL4A1/2*, *MCAM*, and *SEMA3F* are involved in cell adhesion and maintenance of extracellular matrix integrity. Interestingly, apelin (*APLN*) and its cognate receptor APJ (*APLNR*), which are known to play important roles in angiogenic sprouting [16], were among the top contributors (Figure 3B). To better understand the roles of the top candidate genes in response to anti-VEGF inhibition, we decided to focus on *APLN* and *APLNR*, both of which were found to be upregulated in the stroma from the resistant tumors compared to sensitive tumor stroma (Figure 3D, 3E).

### Validation of genes that contribute to resistance to anti-VEGF treatment

**Figure 4:**
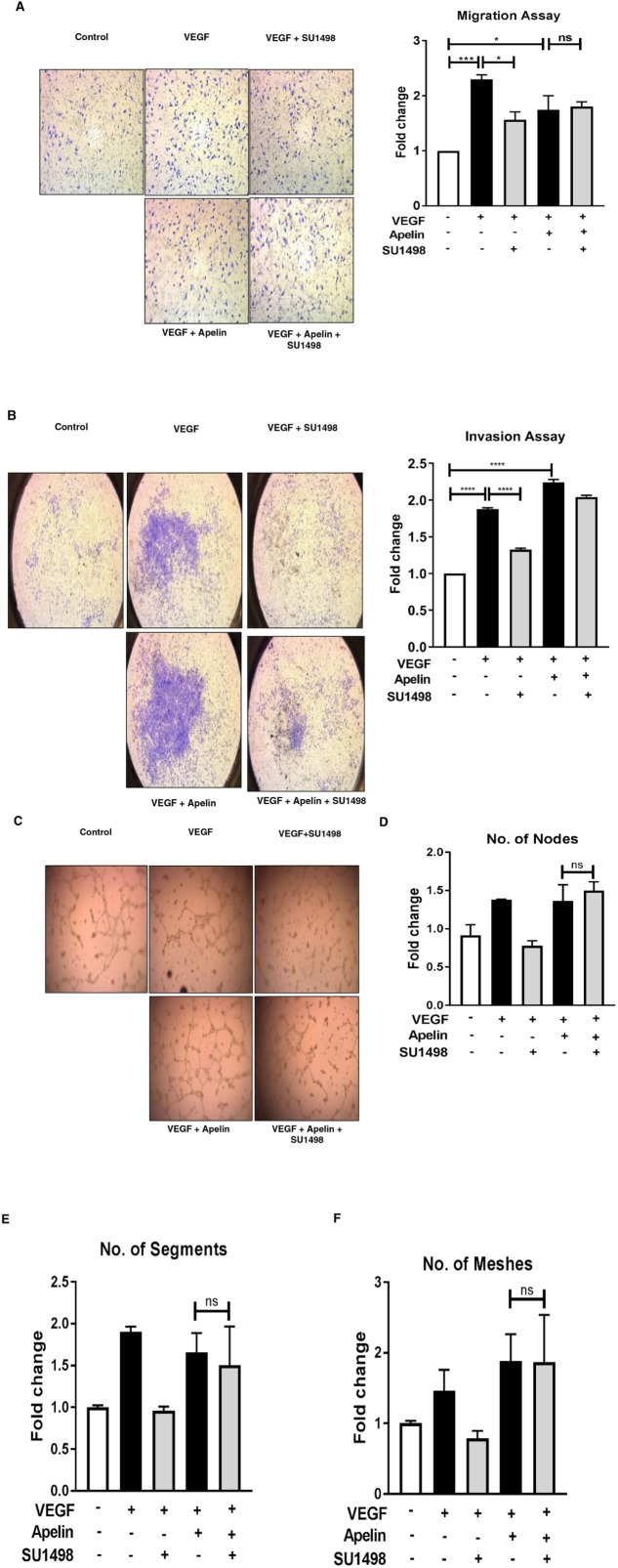
Apelin/APJ pathway contributes to reduced response to anti-angiogenic treatment in endothelial cells **(A)** Representative 6 h-transwell migration assay in EaHY.926 cells and its quantification. **(B)** Representative 24 h-transwell invasion assay performed in EaHY.926 cells and its quantification. **(C)** Representative 16 h-tube formation assay in EaHY.926 cells and **(D-F)** quantification of various tube formation parameters. 10 ng/mL and 50 ng/mL of apelin-13 and VEGF respectively were used. SU1498 was used in the concentration range of 0.25-1.5 μM, depending on the assay. Results obtained from ≥3 independent experiments (Mean±SEM). Statistical analysis was performed using one-way ANOVA followed by Tukey’s post hoc test in **B., D.** ^*^P<0.05; ^**^P<0.01; ^***^P<0.001; ^****^P<0.0001; ns: not significant.

The apelin/APJ pathway is known to play important physiological roles in regulating angiogenesis, energy metabolism, and fluid homeostasis [17]. To determine whether upregulation of apelin and its receptor contributes to reduced response to anti-VEGF therapy, we used two different endothelial cell lines (EaHY.926 and b.End3) that endogenously express high levels of APJ compared to normal ovarian epithelial or cancer cells (Supplementary Figure 3A). We first tested whether these cell lines were sensitive to exogenous addition of apelin (to activate the pathway), and found that 10 ng/ml apelin is as effective as 50 ng/ml VEGF in promoting endothelial cell proliferation in b.End3 cells (Supplementary Figure 3B). For our validation studies, we tested the effect of SU1498 on various *in vitro* phenotypes of endothelial cells in the presence of activated apelin pathway. SU1498 was chosen since it is a specific and potent VEGFR2 inhibitor that has shown efficacy both *in vitro* and *in vivo* [18]. VEGF was added to the cells as a control, and to mimic the presence of angiogenic factors *in vivo*. As expected, we found that addition of VEGF increased the migration (Figure 4A) and invasion (Figure 4B) of EaHY.926 cells. Tube formation as measured by various parameters, including the number of nodes, segments, and meshes, was also increased in the presence of VEGF (Figure 4C-4F). Each of these phenotypes was inhibited when SU1498 was added (Figure 4A-4F). Interestingly, the addition of apelin in the presence of VEGF either maintained high levels of these phenotypes (compared to control), or increased them to levels higher than those observed with VEGF alone. More importantly, addition of apelin and hence, the activated APJ pathway rendered SU1498 ineffective, as the drug was unable to revert any of the VEGF-induced pro-angiogenic phenotypes to control levels (Figure 4A-4F). The same phenomenon was observed in the b.End3 cells, in the cases of migration (Supplementary Figure 4A), invasion (Supplementary Figure 4B), and tube formation (Supplementary Figure 4C-4F). Together, these data indicate that activated apelin/APJ pathway in endothelial cells may contribute to decreased efficacy of anti-VEGF therapeutics.

### High apelin/APJ expression correlates with worsened prognosis in ovarian cancer patients treated with bevacizumab

**Figure 5:**
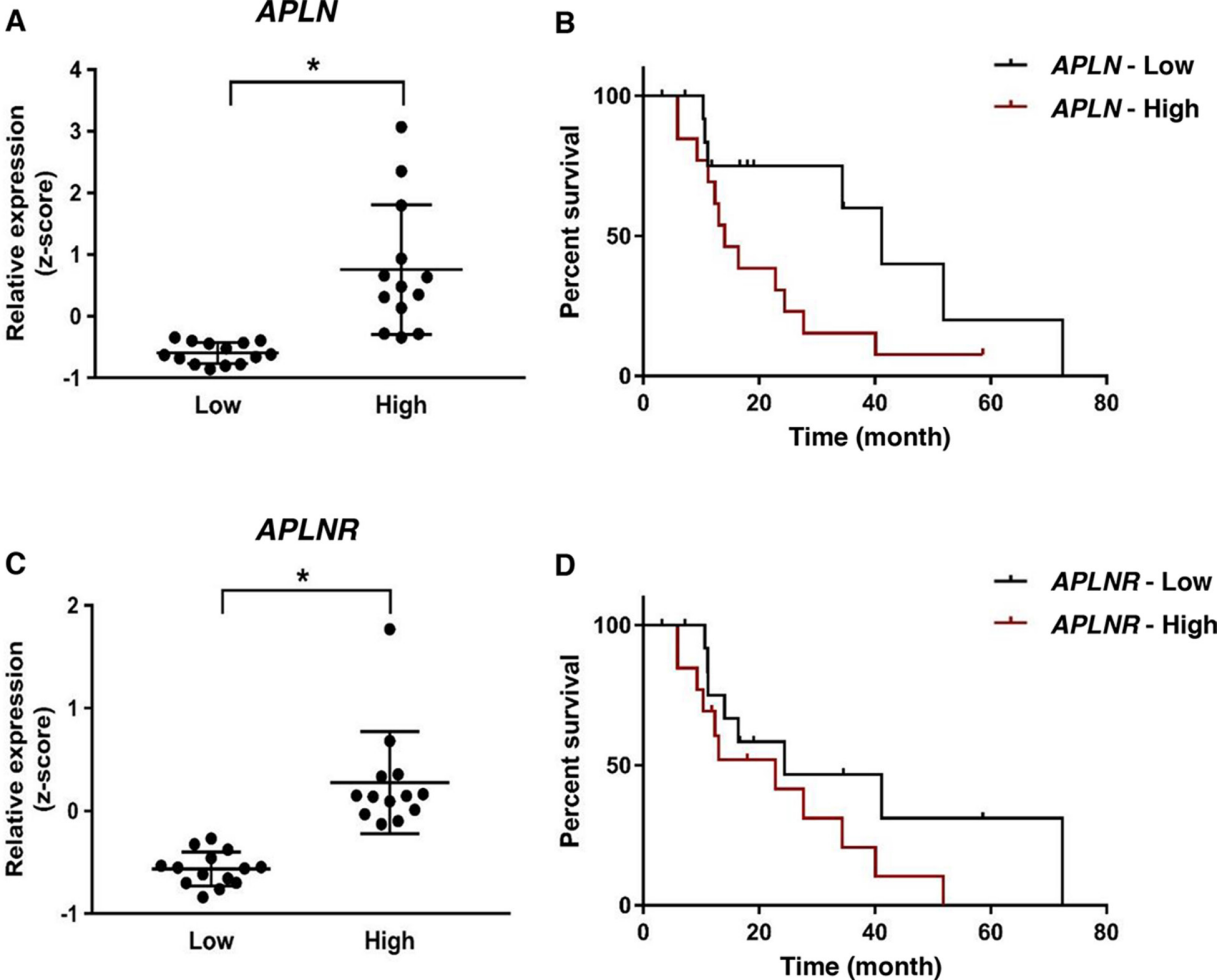
*APLN* overexpression correlates with worsened prognosis in ovarian cancer patients treated with bevacizumab **(A)** *APLN* expression in patients in APLN-low and APLN-high groups. **(B)** Kaplan-Meier analysis of disease-free survival (DFS) in patients in APLN-low and APLN-high groups (Median DFS of 14.1 vs. 41.2 months; P = 0.05). **(C)** *APLNR* expression in patients in APLNR-low and APLNR-high groups (P < 0.001). **(D)** Kaplan-Meier analysis of DFS in patients in APLNR-low and APLNR-high groups (P = 0.22). Statistical analysis was performed using Two-tailed unpaired t-test for gene expression levels in **A., C** and Log-rank test was used for statistical analysis of survival outcome in **B., D.**

To assess the role of increased apelin and/or APJ expression in response of ovarian cancer patients to anti-angiogenic therapy, we evaluated the differences in disease-free survival (DFS) in patients treated with bevacizumab, who expressed variable levels of the genes. Clinical and gene expression data were retrieved from The Cancer Genome Atlas (TCGA) project. Patients were classified as *APLN* or *APLNR*-high or -low using the median value of gene expression z-score as a cut-off. We first confirmed the difference in expression levels between the groups (Figure 5A, 5C). The analysis revealed that patients with high expression of *APLN* had remarkably shorter DFS compared to those expressing lower levels of the gene (median DFS of 14.1 vs. 41.2 months respectively, Figure 5B). While there was a similar trend in the case of *APLNR*, the results did not reach statistical significance (Figure 5D). These results confirm that in ovarian cancer patients treated with bevacizumab, high expression of the pathway, especially *APLN* correlates with worsened prognosis.

## DISCUSSION

Ovarian cancer, a deadly disease due to its late-stage diagnosis, relies heavily on angiogenesis for tumor growth and progression [19]. While anti-VEGF therapies have shown clinical benefits, the rapid development of resistance and hence eventual tumor progression has been a major hurdle in clinical settings. In light of this, there is an urgent need to identify biomarkers that indicate early emergence of resistance to anti-VEGF therapy. It would also be useful to identify molecules that allow for selection of patients most likely to benefit from anti-VEGF therapies, given the high treatment cost and substantial side effects of current treatment strategies. While several clinical studies have evaluated some circulating angiogenic molecules, such as VEGF, PLGF, and sVEGFR as potential biomarkers, their application has shown mixed results [20]. Another important step forward would be to ascertain mechanisms by which tumors mediate resistance to anti-angiogenic drugs. Identification of such novel targets would enable the use of combination therapies to counteract the existing bypass mechanisms.

Traditionally, resistance mechanisms studies have used *in vitro* culturing of drug-sensitive cancer cell lines with increasing drug concentrations until the emergence of resistant clones, as a model to elucidate their genetic and biochemical alterations [21]. However, the same approach is not applicable to anti-angiogenic drugs, since the cancer cells may not be their primary target. Studying resistance mechanisms of non-cancer cell targeting agents is thus challenging due to lack of appropriate model systems that closely mimic the changes in both, tumor microenvironment and cancer cells. Also, researchers have proposed that the developed resistance may be due to either, or both, the tumor and the non-tumor compartments [22]. Therefore in the present study, we developed an *in vivo* model of adaptive resistance to anti-VEGF therapy that closely mimics clinical resistance in ovarian cancer [10, 23, 24]. In our xenograft model, VEGF-targeted agents exhibited modest and transient benefits, followed by the development of resistance to the drug as seen by tumor regrowth. The transient effect of anti-VEGF therapy is thought to be largely due to a redundancy in angiogenic signaling or, alternately, due to pre-existing compensatory angiogenic pathways that are stimulated when continuous inhibition of the VEGF pathway occurs [10, 25–27].

To identify potential pathways mediating the observed anti-VEGF resistance, we profiled the changes in gene and protein expression as tumors progressed from the initial responsive phase to becoming refractory to the drugs. We used two different anti-angiogenic drugs, bevacizumab and sorafenib, to gain insight into the mechanisms of resistance against a class of VEGF anti-angiogenics. Our xenograft tumor models also provided a significant advantage in distinguishing the origin of those genes (human cancer vs. mouse stromal cells) in order to identify the source of resistance. We ensured the clinical and biological significance of our candidate genes by overlapping our list with the core response markers to VEGF signaling inhibition that have been previously validated in human cancers. Brauer et al. [15] reported that this cluster of VDV genes showed both marked downregulation in tumor stroma in response to anti-VEGF treatment, and upregulation in response to VEGF stimulation *in vivo*; in various preclinical tumor models and human tumor biopsies, with various treatment durations. This consistency in their regulation observed across various studies suggested that these genes may represent the core response markers to vascular signaling. Given this, our identified distinct signature of genes and proteins from the treatment-resistant tumors may identify resistance and/or treatment response markers and hence, therapeutic strategies to overcome this resistance.

Using gene ontology and pathway analysis, we identified both well-known and novel genes that are associated with anti-VEGF resistance. We found activation of various pro-angiogenic factors and signaling pathways in resistant tumors that other groups have also reported, supporting the validity of our models. Those molecules include fibroblast growth factors (FGF) 1 and 2, hepatocyte growth factor, and angiopoietins [27–30]. While bevacizumab suppresses tumor growth via inhibition of tumor-derived VEGF in xenograft mouse models [31], residual angiogenesis and tumor growth due to murine VEGF produced by host stroma has been reported [32]. We thus cannot negate the effect of host-derived VEGF in the bevacizumab-treated group. Nevertheless, we identified several other molecules, such as caveolin-1, as being the most upregulated proteins in both BR and SR tumors, the roles of which have been underappreciated in anti-VEGF resistance. While caveolin is critical for angiogenic response to exogenous stimuli, such as bFGF and VEGF [33, 34], its role in cancer is still unclear. Studies show that its activity is context-dependent; functioning as a tumor promoter or suppressor depending on tumor type, stage, and localization (tumor cell or stroma) [35, 36].

Among the pathways identified, we validated the role of apelin and its receptor APJ *in vitro*, since both the ligand and receptor were upregulated in the stroma of resistant tumors. Several studies have demonstrated that anti-angiogenic therapies cause an increase in hypoxia because of reduction in vessel perfusion [37, 38]. This antiangiogenic therapy-induced hypoxia within tumor microenvironment increases HIF1α levels (master regulator of hypoxia) resulting in upregulation of various alternative angiogenic factors. HIF1α increases the levels of apelin which activates the APJ pathway [39, 40]. We hypothesize this as the mechanism for the observed upregulation and activation of the apelin/APJ pathway in anti-VEGF resistant tumors. Our findings demonstrated that endothelial cells do not respond to anti-VEGF therapeutics in the presence of activated apelin signaling. In parallel with our studies, other groups have shown that inhibition of the apelin/APJ pathway improves efficacy of established anti-angiogenic treatments in glioblastoma, and prevents the metastasis associated with anti-angiogenic therapy in breast carcinomas [41, 42]. Importantly, we showed that in patients treated with bevacizumab, increased apelin expression correlated with worsened prognosis. This finding is not limited to ovarian cancer, since more recently and further validating our results, a study of colorectal cancer presented apelin as a potential biomarker in patients that do not respond to bevacizumab treatment [43]. Moreover, the non-angiogenic roles of this pathway are slowly being recognized in cancer, as we recently established the APJ pathway as an important regulator of tumor progression and metastasis in ovarian cancer, independent of its role in increasing angiogenesis [44]. Given that it also causes refraction to anti-VEGF therapeutics, inhibition of this pathway may lead to more complete eradication of the tumor. Since multiple pathways are activated upon VEGF inhibition, it is likely that other pathways identified in this study also contribute to the resistance phenotype. These pathways may provide several avenues for novel combination strategies.

In summary, we have used *in vivo* tumor models that gain adaptive resistance to VEGF-targeting therapeutics to discover a unique molecular signature associated with the anti-VEGF resistance phenotype. These pathways may function as important alternative angiogenic signaling pathways in the presence of VEGF blockade. The present study has thus paved the way for the development of new combination or sequential treatment strategies that may help to counteract the resistance mechanisms.

## MATERIALS AND METHODS

### Reagents and cell culture

Human ovarian cancer cells SKOV-3 and mouse brain endothelial cells bEND.3 were purchased from American Type Culture Collection (ATCC). Primary Human Umbilical Vein Endothelial Cells (HUVEC) and EaHY.926 cells were a generous gift from Dr. Florea Lupu (Oklahoma Medical Research Foundation, Oklahoma City, OK). The cell lines were profiled via short tandem repeat profiling to confirm their identity before receipt. SKOV-3 cells were cultured in McCoy’s 5A medium supplemented with 10% fetal bovine serum (FBS), 100 IU/mL of penicillin, and 100 μg/mL of streptomycin. EaHY.926 cells were cultured in DMEM medium supplemented with HEPES, HAT (hypoxanthine, aminopterin, and thymidine), and FBS. The bEND.3 cells were cultured in DMEM medium supplemented with 10% FBS, 100 IU/mL of penicillin, and 100 μg/mL of streptomycin. HUVEC cells were cultured in endothelial cell growth media (Cell Applications, Inc; catalog no.: 211-500). Cells and media were periodically tested for mycoplasma using the MycoAlert™ Mycoplasma Detection Kit (Lonza), and, if found positive, older freezes of mycoplasma-free cells were used. Sorafenib was obtained from LC laboratories (Woburn, MA). Bevacizumab (Avastin^®^) was obtained from the Stephenson Cancer Center Pharmacy (Oklahoma City, OK). Apelin-13 was purchased from Bachem (H-4568), recombinant VEGF from Peprotech (450-32, 100-20C), and SU1498 from Sigma-Aldrich (SML1193). Drug solutions were prepared according to the manufacturer’s protocol.

### *In vivo* models

All animal studies were performed in accordance with the guidelines and protocols approved by the Institutional Animal Care and Use Committee (IACUC) at the University of Oklahoma Health Sciences Center (OUHSC). We have previously described the generation of preclinical sorafenib-resistant xenograft tumors using human ovarian cancer cells [11]. Similarly, bevacizumab-resistant xenograft tumors were established. Briefly, SKOV-3 cells (5 × 10^6^) in PBS were injected subcutaneously in the right flanks of 6-week-old female athymic nude mice (Charles River Laboratories, Inc., through NCI, Frederick, MD). When tumors attained a volume of approximately 80 mm^3^ (~32 days post-tumor cell implantation), treatment with saline, sorafenib, or bevacizumab was initiated. Sorafenib (30 mg/kg) was administered orally once a day. Bevacizumab (10 mg/kg) was administered intraperitoneally twice a week. Mice were treated with these drugs for up to 8 weeks. Xenograft tumors that progressed with a long-term trend towards continued growth (i.e., > 50% of the initial tumor volume at the start of treatment) after an initial response to treatment were considered phenotypically resistant to treatment (BR: bevacizumab-resistant; SR: sorafenib-resistant). In contrast, tumors that remained responsive to the treatment were considered treatment-sensitive (BS: bevacizumab-sensitive; SS: sorafenib-sensitive). Tumor tissue was collected at the time of euthanasia for the transcriptome, proteome, and immunohistochemical analyses described below.

### RNA-seq analysis

The RNA-seq analysis was performed to profile transcriptome changes in tumor samples (*n*=5/group) from each of the following groups: pre-control (tumors before treatment initiation), control (vehicle-treated tumors at the study endpoint), BS, BR, SS, and SR. Total RNA was extracted from tumors using a Microarrays Total RNA Isolation Kit (Thermo Fisher Scientific, Grand Island, NY). RNA quantity was determined using Agilent Nanodrop. Quality was assessed on an Agilent 2100 Bioanalyzer, according to the manufacturer’s instructions. Sequencing was performed using an Illumina MiSeq sequencer and an Illumina TruSeq RNA v2 sample preparation kit and protocols (Illumina, Inc.). One microgram of total RNA was used per sample for library construction per Illumina’s TruSeq RNA protocols. Equimolar amounts of three whole transcriptome samples were pooled. Six pM of the pooled samples were analyzed on an Illumina MiSeq sequencer using paired-end 2x150-bp sequencing. Per Illumina’s recommendation, 5% phiX library was spiked into the library pool prior to loading for quality control purposes. Approximately 30 million reads were collected for each run. Analysis was performed using Perkin Elmer’s GeneSifter software. The data were aligned to the H. sapiens and M. musculus referenced genomes, which allowed differentiation of human-originated genes (i.e., tumor-originated genes) from mouse-originated genes (i.e., stroma-originated genes) in the tumor microenvironment. This data will be made available upon request.

### Bioinformatics data analysis

We performed exploratory analysis to identify differentially expressed genes and/or pathways that correlate with resistant tumor phenotypes. Data were log2 transformed and replicate measurements were averaged. Based on Euclidean distance, heat-map and hierarchical clustering was performed on the differentially expressed genes among all treatment groups. Principal Component Analysis (PCA) was performed based on covariance matrix on candidate genes associated with response phenotypes and treatment types. The first two principal components explained 70% of the variance. The Ward’s Clustering algorithm was applied to the PCA transformed data [45]. The clustering result was evaluated by comparison to an objective external criterion, which is the assigned group (BS, BR, SS, or SR) of each sample. The transcriptional responses of top candidate genes were analyzed using Kernel Density estimation [46]. The analysis of the gene transcripts was processed by R/Bioconductor [47]. Functional and canonical pathway analyses were performed using QIAGEN's Ingenuity^®^ Pathway Analysis software (IPA^®^, Ingenuity Systems, https://www.ingenuity.com). Differentially expressed genes were uploaded to the Ingenuity website for analysis. Gene expression data were obtained from an ovarian cystadenocarcinoma study publicly available in TCGA database. Z-score normalized RNASeqV2 data for *APLN* and *APLNR* and the associated clinical data from 27 patients treated with bevacizumab [48] were retrieved from The Cancer Genomic Data Server (CGDS) hosted by the Computational Biology Center at Memorial-Sloan-Kettering Cancer Center using the R software, package “cdgsr”. The median cut-off was applied to classify low and high expression. Disease-free survival (DFS) was assessed for treatment outcome.

### Immunohistochemistry

Antibodies specific to CD31 (Abcam, catalog no.: ab28364) and Ki-67 (Abcam, catalog no.: ab16667) were used to determine microvessel density and tumor cell proliferation, respectively. Immunohistochemistry staining was performed on 5-µm formalin-fixed, paraffin-embedded serial mouse xenograft tissue sections. The sections were incubated overnight at 4ºC with primary antibodies to CD31 (1:50) and Ki-67 (1:200), followed by 30 min incubation with a peroxidase-conjugated secondary antibody using the Vector Impress kit (Vector Laboratories, Burlingame, CA). Quantification of positively stained regions in slides was performed using the RGB image profiling method with Image J software for Ki-67 and Microvessel Algorithm in the Aperio software Imagescope for CD31.

### Reverse-phase protein array (RPPA)

RPPA analysis was performed to profile changes in protein levels in tumor samples isolated from five groups: control (vehicle-treated tumors at the study endpoint), BS, BR, SS, and SR. The RPPA assay was performed by the Functional Proteomics RPPA Core Facility at MD Anderson Cancer Center. The detailed protocol is available online at https://www.mdanderson.org/ [49]. Data were normalized for protein-loading correction factor and analyzed. The reported values represent an average of five different tumors for each group.

### Endothelial cell proliferation assay

The effects of apelin and VEGF on bEND.3 cell proliferation were studied using a BrdU incorporation assay. Five thousand b.End3 cells were seeded in a 96-well plate. The cells were incubated at 37°C overnight before treatment with apelin-13 (10 ng/ml) or VEGF (50 ng/ml). Cell proliferation was measured with a BrdU incorporation assay, following the manufacturer’s protocol (Roche, Basel, Switzerland).

### Tube formation assay

Plates (96-/24-well) were coated with Matrigel^®^ Growth Factor Reduced Basement Membrane Matrix (Corning, NY). EaHY.926 (15,000 cells/well) and bEND.3 cells (140,000 cells/ well) were plated in medium (2% FBS) and incubated for 16 h and 5.5 h, respectively. Apelin-13 (10 ng/mL) and VEGF (50 ng/mL) were added at the time of plating. SU1498 was used in the concentration range of 0.25-0.5 μM. The networks formed were fixed with 4% paraformaldehyde and imaged using a bright field microscope (Leica). The extent of tube formation was quantified using the Angiogenesis analyzer tool in ImageJ.

### Migration assay

Migration assays were performed using 8-μm Transwell cell culture inserts (BD Falcon, 353097).

EaHY.926 (30,000 cells/well) were plated on a Transwell filter in serum-free medium and were allowed to migrate towards medium containing 10% FBS for 6 h. For bEND.3 cells, 40,000 cells were plated on the insert in medium containing 2% FBS and incubated overnight. The following day, cells were allowed to migrate towards medium containing 10% FBS for 6 h. The ligands apelin-13 (10 ng/mL) and VEGF (50 ng/mL) were added in the bottom chamber. SU1498 in the concentration range of 0.25-0.5 μM was added to the insert. At the end of the experiment, cells from above the membrane were wiped with cotton swabs, and migrated cells at the bottom were fixed with 10% formalin and stained with 0.05% crystal violet (CV). Cell migration was quantified by counting cells using a bright field microscope (Leica) and Image J.

### Invasion assay

Invasion assays were performed using 8-µm Transwell cell culture inserts (BD Falcon), after coating filters with 1:20 diluted Matrigel (Fisher, CB40230) in serum-free medium. EaHY.926 cells (100,000/well) were plated on the Matrigel in medium containing 2% FBS and were allowed to invade toward medium containing 10% FBS for 24 h. For bEND.3 cells, 40,000 cells were plated on the insert in medium containing 2% FBS and were incubated overnight. The following day, cells were allowed to invade towards medium containing 10% FBS for 48 h. The ligands apelin-13 (10 ng/mL) and VEGF (50 ng/mL) were added to the bottom chamber. SU1498 in the concentration range of 1–1.5 μM was added to the insert. Cell invasion was analyzed similar to Transwell migration assays.

### RT-qPCR

Total RNA was extracted from cell pellets using E.Z.N.A.® HP Total RNA kit (Omega Bio-Tek, Norcross, GA), and reverse-transcribed to cDNA using Maxima cDNA synthesis kit (Thermo Fisher) from 0.5-1 ug of mRNA. qRT–PCR assays were performed using ssoFast Evagreen supermix (BioRad) in CFX96^TM^ Real-Time PCR Detection Systems (BioRad, Hercules, CA). The primers used were: APJ, forward: 5′-TGGTGCTCTGGACCGTGTTT-3′; reverse: 5′-TGAGGTAGCTGCTGAGCTTG-3′; GAPDH, forward: 5′–GACCCCTTCATTGACCTCAAC–3′; reverse: 5′–CTTCTCCATGGTGGTGAAGA–3’. The thermal reaction program was: 30 sec at 95°C, 40 cycles of 5 sec at 95°C, and 5 sec at 55°C. APJ mRNA level relative to GAPDH mRNA was calculated using the ΔΔCt method [50].

### Statistical analysis

GraphPad Prism version 7.0 for Windows (GraphPad Software, La Jolla, CA) was used for all statistical analyses. Two-tailed unpaired Student’s *t*-test was used to compare pairs of conditions. One-way Analysis of Variance (ANOVA) non-parametric followed by Tukey’s post hoc test was used to compare more than two conditions. For bioinformatics data analysis, ANOVA adjusted for multiple comparisons using the Benjamin and Hochberg False Discovery Rate (FDR) method was used. Fischer’s exact test was used for IPA analysis. Log-Rank test was used to determine statistical significance of the differences between two groups for TCGA datasets. Student’s *t*-test with the false discovery rate (FDR)-adjusted *p* values using the Benjamini-Hochberg method was used to determine the significance for RPPA. A *P* value of <0.05 denoted statistical significance.

## SUPPLEMENTARY MATERIALS AND FIGURES



## Abbreviations

VEGF(R): Vascular Endothelial Growth Factor (receptor)
TKI: tyrosine kinase inhibitor
BR/SR: Bevacizumab/Sorafenib resistant
BS/SS: Bevacizumab/Sorafenib sensitive
MVD: Microvessel density
VDV: VEGF dependent vasculature
IPA: Ingenuity pathway analysis
Apln(r): Apelin (receptor)
DFS: Disease free survival
