# Supplementary material for: Identification of signature genes associated with therapeutic resistance to anti-VEGF therapy
Source: Oncotarget. 2020 Jan 7;11(1):99–114. doi: 10.18632/oncotarget.27307 (PMC6967771; doi:10.18632/oncotarget.27307)
Supplement: Supplementary file 1 [file oncotarget-11-99-s001.pdf]

Identification of signature genes associated with therapeutic resistance to anti-VEGF therapy

SUPPLEMENTARY MATERIALS

REFERENCES

1. Devapatla B, Sharma A, Woo S. CXCR2 Inhibition Combined with Sorafenib Improved Antitumor and Antiangiogenic Response in Preclinical Models of Ovarian Cancer. PLoS One. 2015; 10: e0139237. https://doi.org/10.1371/journal.pone.0139237. [PubMed]

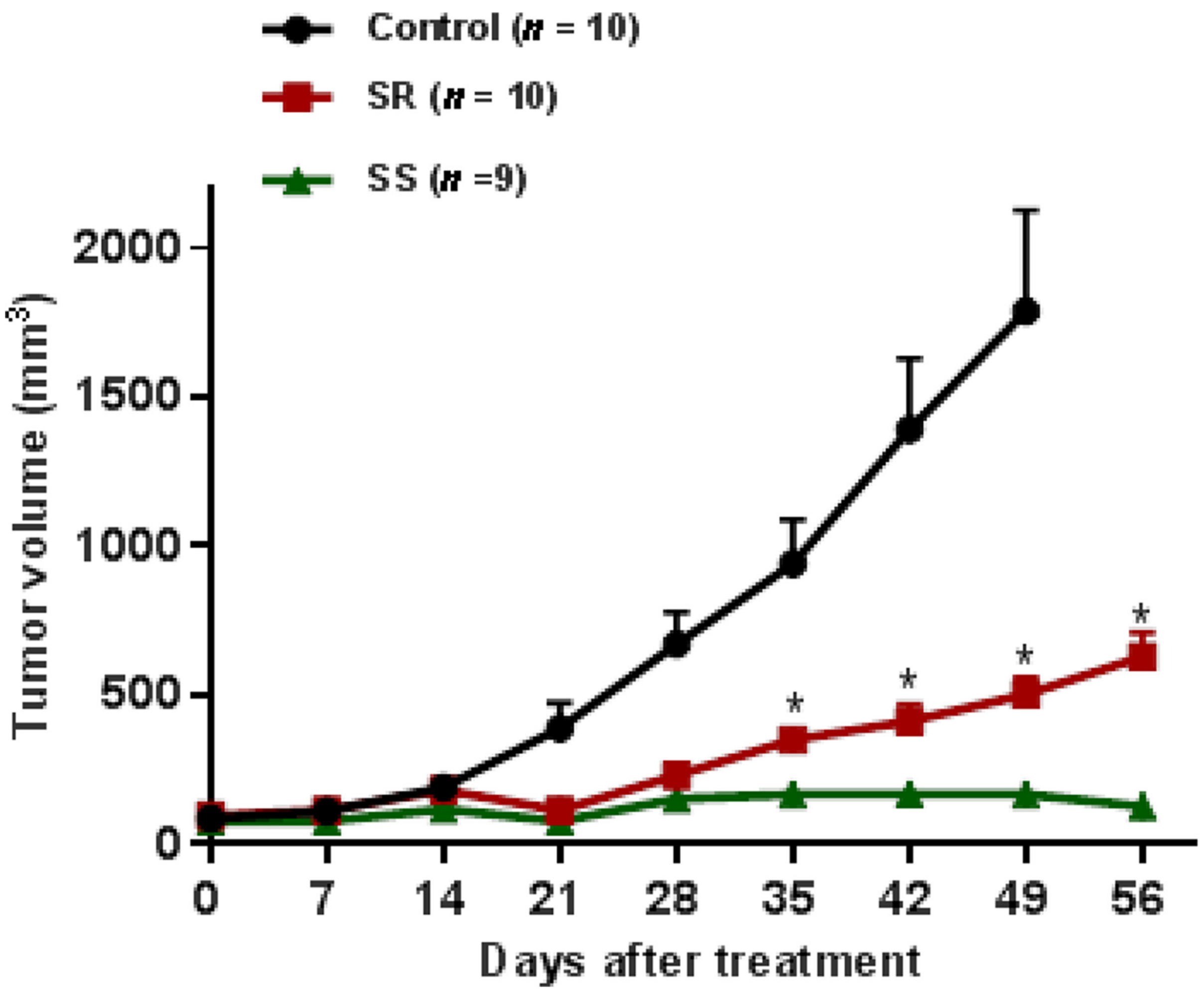

Supplementary Figure 1: Phenotypic resistance to sorafenib in a SKOV-3 xenograft mouse model of ovarian cancer. SKOV-3 xenografts were treated for 8 weeks with 30 mg/kg daily sorafenib via oral gavage. Controls (n=10) were treated with the corresponding treatment vehicle alone. Tumor volumes were measured twice a week and are represented as mm3 ± SEM. In the sorafenib-treated group, 10 of 19 mice developed resistance to the treatment. A significant difference in tumor volume (* $P < 0.05$) was observed between the resistant and sensitive groups, starting at week 5 of treatment. SS = sorafenib-sensitive; and SR = sorafenib-resistant. **Figure is taken from [1].

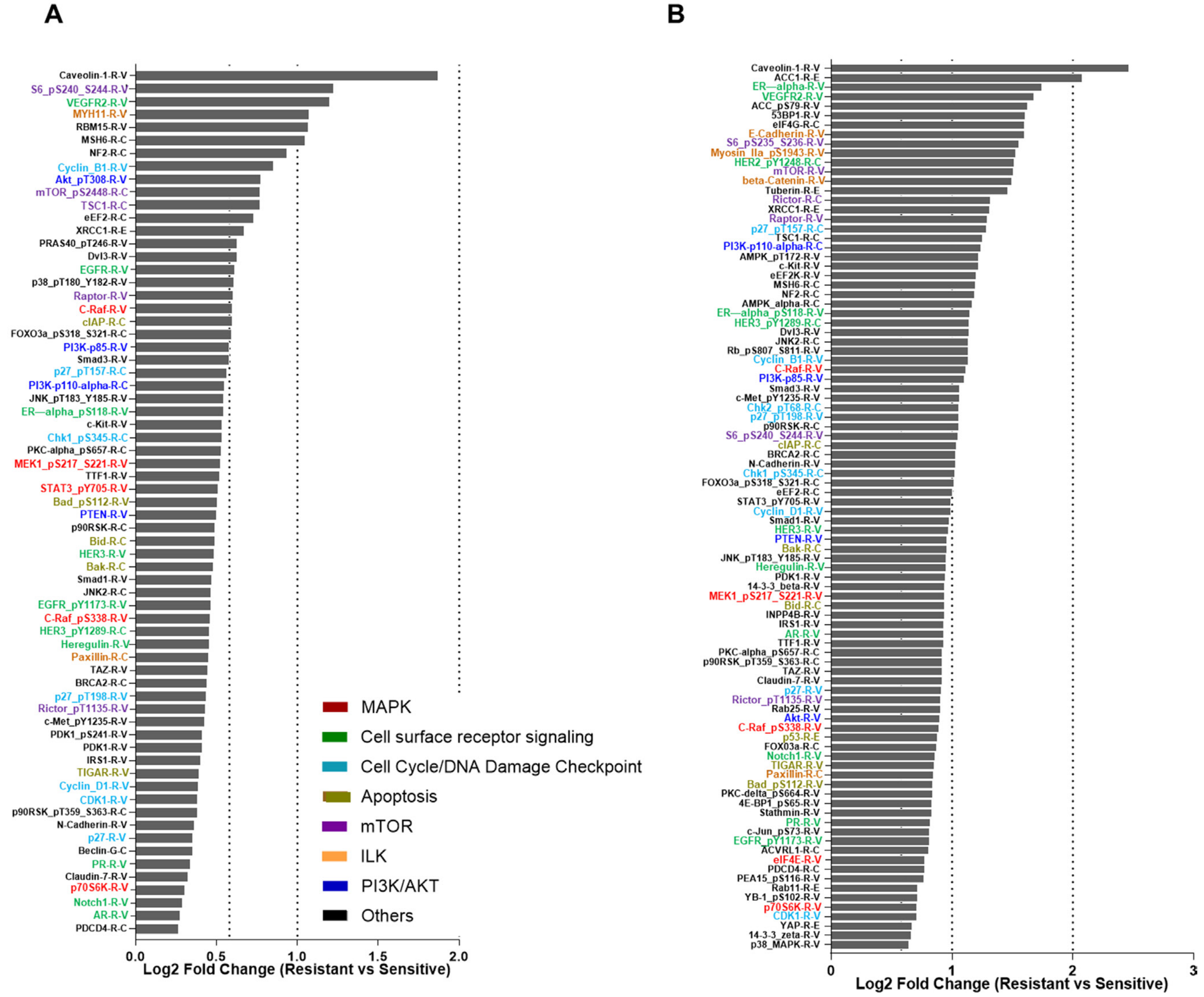

Supplementary Figure 2: Protein array analysis confirms upregulated genes from RNA-seq analyses. Bar chart depicts the log2 FC of protein level in **(A)** BR vs BS tumors and **(B)** SR vs SS tumors of all differentially expressed proteins (FDR-adjusted $p < 0.05$). Proteins are color-coded according to their function. Dashed lines depict fold change of 1.5, 2 and 4.

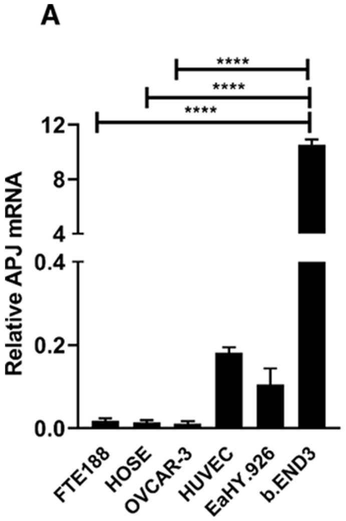

Cell line	Mean Ct values
HOSE	36.24
FTE188	37.97
OVCAR-3	38.36
HUVEC	33.56
EaHY.926	37.07
b.END3	30.15

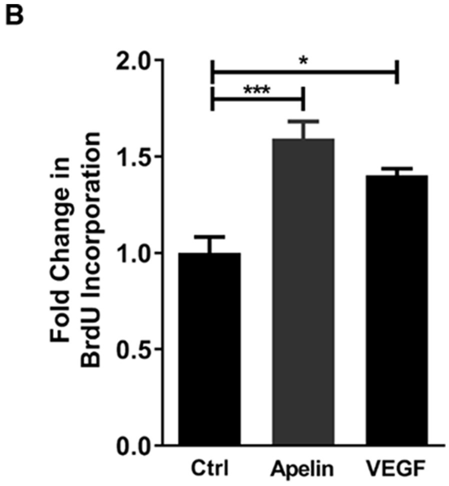

Supplementary Figure 3: Apelin/ APJ pathway promotes endothelial cell proliferation. **(A)** qRT-PCR for APJ expression in fallopian tube epithelial cells (FTE188), human ovarian surface epithelial cells (HOSE), ovarian carcinoma OVCAR-3 cells, human umbilical vein endothelial cells (HUVEC), b.END3, and EaHY.926 cells and the corresponding Ct values. **(B)** Enhanced mitogenic effect in b.END3 cells treated with apelin 10 ng/ml or VEGF 50 ng/ml for 48 hours. Results obtained from 3 independent experiments (mean ± SEM). Statistical analysis performed using one-way ANOVA followed by Tukey's post-hoc test for **(B)**. *$P<0.05$, ***$P<0.001$.

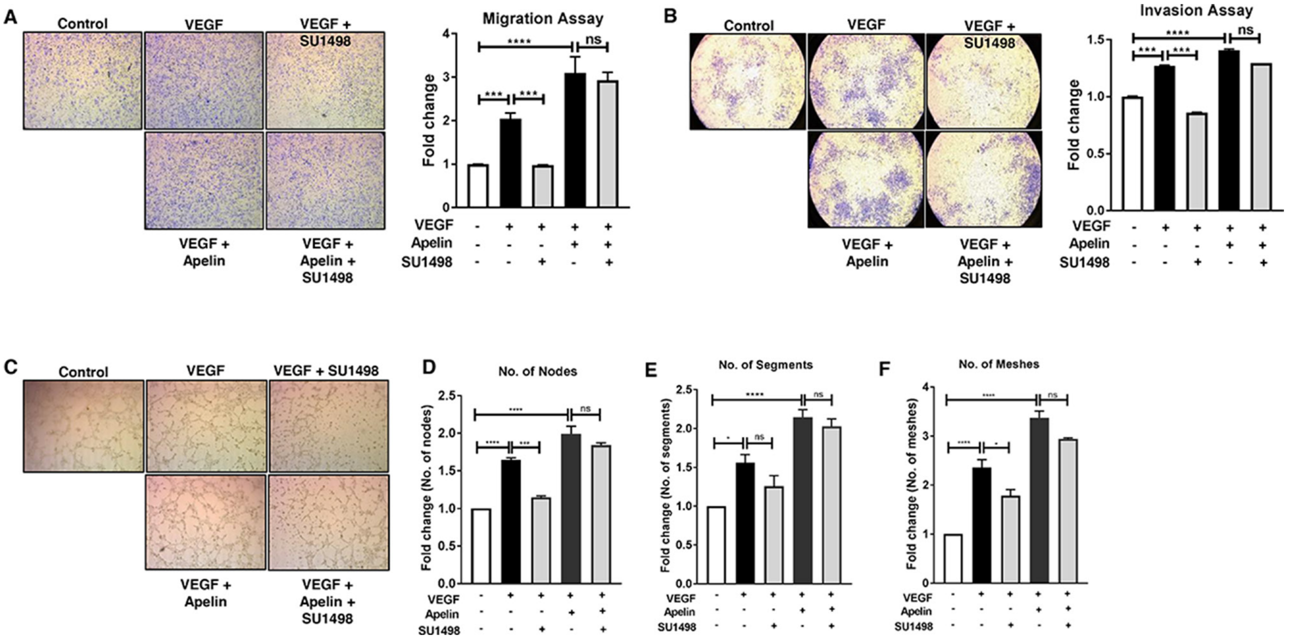

Supplementary Figure 4: Apln/Aplnr pathway contributes to reduced response to anti-angiogenic treatment in b.END3 cells. **(A)** Representative 6 h-transwell migration assay and **(B)** its quantification. **(C)** Representative 24 h-transwell invasion assay and (**D**) its quantification. **(E)** Representative 5.5 h tube formation assay and **(F–H)** its quantification. Concentrations of apelin-13 and VEGF used were 10 ng/mL and 50 ng/mL, respectively. SU1498 was used in the concentration range of 0.25-1.5 μM, depending on the assay. Results obtained from ≥3 independent experiments (Mean±SEM). Statistical analysis was performed using one-way ANOVA followed by Tukey's post hoc test in **(B, D, F–H)**. *P<0.05; ***P<0.001; ****P<0.0001; ns: not significant.

Supplementary Table 1: Top 15 most upregulated and downregulated genes that were expressed differently in tumors that were resistant to bevacizumab or sorafenib treatment

Gene	FC (BR/BS)	*P*-value	Gene	FC (SR/SS)	*P*-value
Top upregulated genes					
PCDH11X	5.31	0.003	***SFRP2***	28.07	0.046
ADGRG5	4.63	0.008	***EMX1***	6.98	0.009
LHX1	4.45	0.002	***KRT6C***	6.77	0.048
LINC01600	4.43	0.022	***ACTG2***	6.68	0.029
FAM180A	4.31	0.004	***PPP1R14A***	6.25	0.040
SLC14A2	4.21	0.042	***ITGB6***	6.08	0.017
KRT24	4.12	0.041	***SYNE3***	6.05	3.2E-05
NTNG1	3.97	6.50E-05	***RAET1L***	5.51	0.009
RAET1G	3.85	0.044	***ANKRD36BP1***	5.33	0.034
H19	3.82	0.030	***FILIP1***	5.21	0.003
RPE65	3.76	0.009	***LINGO1***	5.14	0.001
PTGER1	3.72	0.020	***CLIC3***	5.04	0.003
STRA8	3.63	0.001	***BNC1***	5.02	0.021
RGCC	3.62	0.001	***STON2***	4.87	0.011
RIMS1	3.59	0.028	***WNT9A***	4.75	1.3E0-04
Top downregulated genes					
HLA-DRA	-7.66	0.001	***RSAD2***	-14.78	0.017
CLIC5	-6.29	0.013	***CPXM1***	-8.56	0.018
HLA-DRB1	-6.24	0.010	***HLA-DRA***	-8.39	0.033
LOC644936	-5.90	0.015	***MX2***	-7.34	0.003
HLA-DPA1	-4.85	0.031	***HLA-DRB1***	-7.15	0.002
TFF3	-4.56	0.022	***CHI3L1***	-6.62	0.002
HLA-DQB1	-4.48	0.002	***NECAB2***	-6.18	0.036
CLDN5	-4.43	0.028	***TFF3***	-6.12	0.004
CIITA	-4.43	2.57E-04	***C1QTNF9***	-5.51	0.002
GFAP	-4.33	0.017	***CYP27A1***	-5.22	0.003
CDHR2	-4.29	0.003	***CD74***	-5.17	5.69E-04
COL3A1	-3.92	0.003	***CYP21A2***	-5.11	4.8E-04
VTCN1	-3.63	0.038	***OASL***	-5.04	0.019
ROBO2	-3.55	0.013	***BRSK1***	-4.99	0.015
SERPINB3	-3.51	0.003	***DLX4***	-4.96	0.018

The table shows gene symbol, fold change, and P-value of mRNA expression changes in tumors that are resistant or sensitive to bevacizumab or sorafenib. The top 15 upregulated and downregulated genes are shown. FC = fold change; SR = sorafenib-resistant; SS = sorafenib-sensitive; BR = bevacizumab-resistant; BS = bevacizumab-sensitive.

Supplementary Table 2: Top 13 most upregulated and downregulated genes that were expressed differently in stroma resistant to bevacizumab or sorafenib treatment

Gene	FC (BR/BS)	*P*-value	Gene	FC (SR/SS)	*P*-value
Top upregulated genes					
Csrp3	13.53	0.005	***Ednrb***	11.27	0.001
Ntng1	11.08	8.193E-05	***Adamts12***	11.03	0.017
Xirp2	10.33	0.024	***Cxcl5***	10.92	0.044
Lmod2	9.07	0.006	***Itm2a***	10.57	0.007
H19	8.30	0.003	***Prss35***	10.54	0.034
Xirp1	7.71	0.047	***Fcrls***	9.47	0.005
Neb	7.24	0.038	***Col11a1***	9.19	0.001
Alox5	7.05	0.023	***Prnd***	8.90	8.08E-07
Retnlg	7.04	0.028	***Aplnr***	8.75	2.35E-04
Trpc1	6.48	0.012	***F13a1***	8.29	0.049
Nlrp12	5.61	7.878E-05	***Tusc5***	8.18	0.001
Slc1a4	5.46	7.968E-06	***Scn7a***	8.11	0.025
Col24a1	5.20	0.017	***Steap4***	7.64	0.021
Top downregulated genes					
Pla2g2d	-29.24	0.045	***Nkg7***	-18.99	0.048
Igh-6	-22.67	0.036	***Ccl22***	-10.68	0.027
Cfd	-14.53	0.029	***Ccl5***	-10.38	0.034
Stac2	-10.87	0.017	***Cd3g***	-9.40	0.012
Dnase1l3	-10.65	0.001	***Tgtp***	-7.78	0.042
Slamf7	-10.17	0.005	***Lax1***	-7.46	0.039
Pdcd1lg2	-9.81	0.044	***Cd27***	-7.45	0.045
Adamdec1	-8.91	0.012	***EG434843***	-7.11	0.003
Adipoq	-8.48	0.037	***Tctex1d4***	-6.97	0.002
H2-Q8	-7.88	0.009	***Ccr7***	-6.49	0.041
Lpl	-7.56	0.002	***AA467197***	-6.45	0.045
Klri2	-7.06	0.031	***Pdcd1***	-5.79	0.035
Cidec	-6.72	0.032			

The table shows gene symbol, fold change, and *P*-value of mRNA expression changes in tumors that are resistant or sensitive to bevacizumab or sorafenib. The top upregulated and downregulated genes are shown. FC = fold change; SR = sorafenib-resistant; SS = sorafenib-sensitive; BR = bevacizumab-resistant; BS = bevacizumab-sensitive.
